# Identifying Senescent Cells That Drive Aging

**DOI:** 10.1093/geroni/igab046.950

**Published:** 2021-12-17

**Authors:** Laura Niedernhofer

**Affiliations:** University of Minnesota, Minneapolis, Minnesota, United States

## Abstract

Cellular senescence is a potent tumor suppressor mechanism. However, the untoward effect is that the accumulation of senescent cells promotes loss of resilience, aging and age-related diseases. One approach to maintaining the benefits of senescence while preventing the negative consequences is senolytic therapies: drugs that do not prevent senescence, but selectively kill senescent cells. Since virtually any type of cell can become senescent, it is important to identify the lineages of senescent cells that are most potent at driving loss of tissue homeostasis and aging. This will enable honing development of senolytics. We used a genetic approach to drive increased genotoxic stress, a potent inducer of senescence, in a tissue specific manner. The impact of this targeted senescence on different organs and cell types will be discussed, identifying a lead target for senolytics.

